# Manipulating cinnamyl alcohol dehydrogenase (CAD) expression in flax affects fibre composition and properties

**DOI:** 10.1186/1471-2229-14-50

**Published:** 2014-02-20

**Authors:** Marta Preisner, Anna Kulma, Jacek Zebrowski, Lucyna Dymińska, Jerzy Hanuza, Malgorzata Arendt, Michal Starzycki, Jan Szopa

**Affiliations:** 1 Faculty of Biotechnology, University of Wroclaw, Przybyszewskiego 63/77, Wroclaw 51-148, Poland; 2 Wroclaw Research Center EIT +, Stabłowicka 147/149, Wroclaw 54-066, Poland; 3 Centre of Applied Biotechnology and Basic Sciences, Faculty of Biotechnology, Rzeszow University, Aleja Rejtana 16, Rzeszow, Poland; 4 Department of Bioorganic Chemistry, Institute of Chemistry and Food Technology, Faculty of Engineering and Economics, Wroclaw University of Economics, Komandorska 118/120, Wroclaw 50-345, Poland; 5 Institute of Low Temperatures and Structure Research, Polish Academy of Sciences, Okólna 2, Wrocław 50-422, Poland; 6 The Plant Breeding and Acclimatization Institute (IHAR) - National Research Institute, Research Division Poznan, ul. Strzeszynska 36, Poznan 60-479, Poland; 7 Linum Foundation, Stabłowicka 147/149, Wroclaw 54-066, Poland

**Keywords:** Cinnamyl alcohol dehydrogenase (CAD), Lignin, Cell wall, Flax fibre, *Linum usitatissimum*, L

## Abstract

**Background:**

In recent decades cultivation of flax and its application have dramatically decreased. One of the reasons for this is unpredictable quality and properties of flax fibre, because they depend on environmental factors, retting duration and growing conditions. These factors have contribution to the fibre composition, which consists of cellulose, hemicelluloses, lignin and pectin. By far, it is largely established that in flax, lignin reduces an accessibility of enzymes either to pectin, hemicelluloses or cellulose (during retting or in biofuel synthesis and paper production).

Therefore, in this study we evaluated composition and properties of flax fibre from plants with silenced *CAD* (cinnamyl alcohol dehydrogenase) gene, which is key in the lignin biosynthesis. There is evidence that *CAD* is a useful tool to improve lignin digestibility and/or to lower the lignin levels in plants.

**Results:**

Two studied lines responded differentially to the introduced modification due to the efficiency of the *CAD* silencing. Phylogenetic analysis revealed that flax CAD belongs to the “bona-fide” CAD family. *CAD* down-regulation had an effect in the reduced lignin amount in the flax fibre cell wall and as FT-IR results suggests, disturbed lignin composition and structure. Moreover introduced modification activated a compensatory mechanism which was manifested in the accumulation of cellulose and/or pectin. These changes had putative correlation with observed improved fiber’s tensile strength. Moreover, *CAD* down-regulation did not disturb at all or has only slight effect on flax plants’ development *in vivo,* however*,* the resistance against flax major pathogen *Fusarium oxysporum* decreased slightly. The modification positively affected fibre possessing; it resulted in more uniform retting.

**Conclusion:**

The major finding of our paper is that the modification targeted directly to block lignin synthesis caused not only reduced lignin level in fibre, but also affected amount and organization of cellulose and pectin. However, to conclude that all observed changes are trustworthy and correlated exclusively to *CAD* repression, further analysis of the modified plants genome is necessary. Secondly, this is one of the first studies on the crop from the low-lignin plants from the field trail which demonstrates that such plants could be successfully cultivated in a field.

## Background

**Figure 1:**
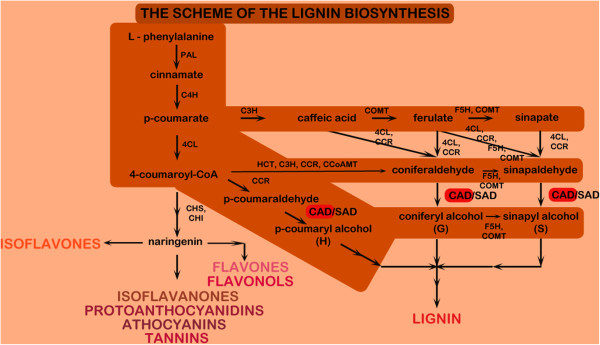
**The simplified scheme of the lignin biosynthesis pathway within the phenylpropanoid route.** 4CL, 4-coumarate:CoA ligase; C3H, p-coumarate3-hydroxylase; C4H, cinnamate 4-hydroxylase; CAD, cinnamyl alcohol dehydrogenase; CCoAOMT, caffeoyl-CoA O-methyltransferase; CCR, cinnamoyl-CoA reductase; CHI, chalcone isomerase; CHS, chalcone synthase; COMT, caffeic acid O-methyltransferase; HCT, p-hydroxycinnamoyl-CoA:shikimate/quinate p-hydroxycinnamoyltransferase; F5H, ferulate 5-hydroxylase; PAL, phenylalanine ammonia-lyase; SAD, sinapyl alcohol dehydrogenase [2,3,5,6,11,14,18].

Lignin is a highly complex, hydrophobic biopolymer, rich in inner hydrogen bonds and/or condensed linkages such as ether and carbon-carbon, biphenyl or biphenyl ether bonds [1]. Monomers that built lignin are: H–, G- and S- unit, which are derivatives of p-coumaryl, coniferyl and sinapyl alcohol respectively [2]. Lignin is synthesized as a branch of the phenylpropanoid pathway (Figure 1). Briefly, p-coumarate is derived from L-phenylalanine and it is further transformed into 4 - coumaryloCoA or caffeic acid (via different routes, see Figure 1). They are the key substrates for next alterations which lead to receiving three aldehydes: p-coumaric aldehyde, coniferyl aldehyde and sinapyl aldehyde which are finally transformed with cinnamyl alcohol dehydrogenase (CAD) to their alcohol derivatives [2-4]. The process occurs in the cytoplasm, near the ER membrane [5]. However, the transport of monolignols to the cell wall, where the polymerization via oxidative radicalization into lignin takes place, is as yet not fully explained [2,6]. Recent development indicates that the transport through the cell membrane might occur via monolignol glucosides [5].

CAD is the final enzyme in the lignin monomers biosynthetic pathway, and its characteristics and properties are described widely [7-10]. More recently, sinapyl alcohol dehydrogenase was discovered and described in aspen (*Populus tremuloides*) [11]. It is an alcohol dehydrogenase related to but distinct from CAD that has high affinity to a sinapyl and not coniferyl aldehyde. However, a number of independent studies proved that CAD and not SAD is responsible for sinapaldehyde reduction during lignification, because in mutant or SAD-deficient plants lignin composition and amount was not altered. Therefore, CAD plays a major role in all three monolignol biosynthesis [12-14]. However, the exact role of *SAD* is still unknown; it might play a role in lignin diversity in gymnosperm having highly specific function or in plant response to the pathogen infection [13].

There is evidence that *CAD* manipulation is a useful tool to improve lignin digestibility and/or to lower the lignin level in plants, which may result in boosting crop properties [15-18].

Earlier research proved that *CAD* down-regulation in tobacco resulted in unchanged lignin level although its structure was altered; the hydroxycinnamic aldehydes were incorporated into lignin. What is more, lignin polymer was more susceptible to digest [15,16,19]. Another researches showed that silencing *CAD* in pine, tobacco and poplar led to changes in the lignin structure and/or their improved digestibility [18,20,21]. Single *CAD* mutation in sorghum caused changes in the phenylpropanoid metabolism, lignin composition and amount, although no changes in plant growth were reported [22]. For switchgrass with down-regulated *CAD* strong reduction of *CAD* transcript was observed, as well as lower lignin level and incorporation of the hydroxycinnamic acids into lignin structure [23]. What is more, transgenic biomass was more susceptible to the cellulose digestion. Similarly, in a model plant, *Arabidpsis thaliana*, strong reduce in CAD activity was connected with incorporation of coniferyl and sinapyl aldehydes into lignin structure [17]. Very recent report on lignin reduction in *Arabidopsis* by double down-regulation of *CAD* and *CCR* (Cinnamoyl CoA reductase) showed that strong lignin reduction affected not only cell wall composition, but also plant growth and development. The latter was demonstrated in sterility and dwarfism of mutant *Arabidopisis*[24].

Flax (*Linum usitatissimum*, L.) is an annual plant, widely grown in temperate climate for its crop, the fibre and seed. Linen fabric was for centuries the major domestic textile in Europe and some other parts of the world and the seed was valuable source of all-purposes oil. However, while there are a few other oil plants cultivated in moderate climate, hardly could be found any other fibrous plant adjusted to growing conditions in a temperate zone.

Flax fibre has bast origin and it is characterized as ligninocellulosic with presence of hemicellulose, pectin, and some metabolites present in relatively small amount, but of great importance [25]. All four biopolymers are tightly bonded, embodied in each other; however, it is worth stressing that so far there is no evidence to correlate fibre property–structure relationship [26].

Retting is a process in which fibre is separated from a stalk and nowadays, the most popular is dew retting, when the flax straw is spread on the ground and then the microorganisms naturally inhabiting the soil are employed to disengage the fibre bundles from the stalk residues [27,28].

Fibre quality highly depends on retting time and the kind of microorganisms, the longer the exposition to atmosphere, the worse fibre’s hue and texture and the more micro defects in the fibre’s structure. Moreover, retting elongation is associated with the progress in the cellulose degradation and weakening fibres’ strength [28,29].

It is worth stressing that recently, in the textile industry the dominant textile is that produced of cotton. However, the cotton fibre is nothing else but pure cellulose. While, thanks to its composition, flax fibre has the highest liquid absorption among natural fibres and it is biologically active, which is by far the greatest advantage over cellulosic cotton fibres.

Unfortunately, in recent decades the popularity of the flax plantations has decreased, not only in Poland, but all over the world [30]. This tendency could be explained by several reasons:

•the fact that in terms of possessing and processing, flax fibre is much more expensive than cotton one;

•hardships associated with flax cultivation (susceptibility to pathogen infection, dependency on weather conditions) and time required to possess fibre after a vegetation season;

•some disadvantages of flax fibre like poor elasticity, unpredictable quality.

Nowadays the new potential applications of flax fibres are under investigation, which may contribute to renewing flax plantations. The spectrum of flax fibre prospective applications is astoundingly wide, from biocomposites, by biofuels, special paper production, bioremediation for biomedical applications [31-39]. However, still it is more science than industry. Unfortunately, to successfully cultivate flax plants and by extension to launch the new products with attractive properties currently cultivated flax varieties are not efficient. There is a need to search for new flax varieties with reduced above-mentioned disadvantages. One of the solutions might be reduction of lignin level in flax, resulting in shortening retting time which should minimalize unpredictability of flax fibre fineness. Additionally, it might lead to improve fibre properties and even more, applicability.

In flax lignin composition and structure is the subject of many research, its amount varies between 3 and 6% of dry weight depending upon environmental factors or flax varieties [3,40-44]. Quite recently, however, it was revealed that flax bast lignin is unusual. Flax is an angiosperm; however, its bast lignin strongly resembles gymnosperm lignin whereas xylem lignin is typical angiosperm. Moreover, in flax, bast cells are lignified in the lowest amount among bast fibers, but on the other hand lignin has the highest ratio of condensed linkages, which practically makes them chemically inactive [40,41,45]. By far, it is largely established that in fibre, lignin ensures stiffness but this results in poor elasticity and wrinkling of linen textile. Moreover lignin elongates retting time as it blocks enzymes access to pectin and hemicellulose, while at the same time, acting as a mechanical barrier, it participates in plant defence against pathogens. Lignin also reduces an accessibility of cellulose to degradation both in the biofuel synthesis and paper production [46-49]. Therefore, reducing lignin level should lead to improvement in fibre quality and by extension properties. The data on reducing lignin level in flax is very limited. Day et al. repressed Caffeoyl CoenzymeA O-Methyl Transferase (CCoAOMT) in flax [3]. The plants obtained in the greenhouse showed lower lignin level (8-18%) which was accompanied by the thinner cell wall and an irregular xylem phenotype. The modified plants had only slight reduction in CCoAOMT and this was accompanied by no changes in plants’ phenotype.

Thus, to enhance flax fibre fineness, competitiveness and applicability we generated flax plants with silenced *CAD* gene and as a result reduced lignin level. Our preliminary research proved that low-lignin flax plants had improved tensile strength [50]. Presently, there is a need of careful analysis of the cell wall composition in the crop of the modified plants, the fibre as well as estimating how the modification affects plant phenotype *in vivo*.

All in all, the aim of this study was to evaluate how silencing *CAD* gene affects flax fibre composition, properties, and also physiology of modified fibrous flax plants (*Linum usitatissimum* L. cv. Nike) grown in the field. In particular, we estimated significance of the *CAD* repression to:

i. the lignin level in fibre;

ii. the composition and structure of cell wall;

iii. the phenotype of modified plants *in vivo*;

iv. the properties of flax fibre.

To the best of our knowledge this is one of the very first studies investigating the composition and properties of the crop (fibre in particular) from modified flax plants.

## Results

### The characteristic and expression of CAD and SAD involved in final monolignol synthesis

**Figure 2:**
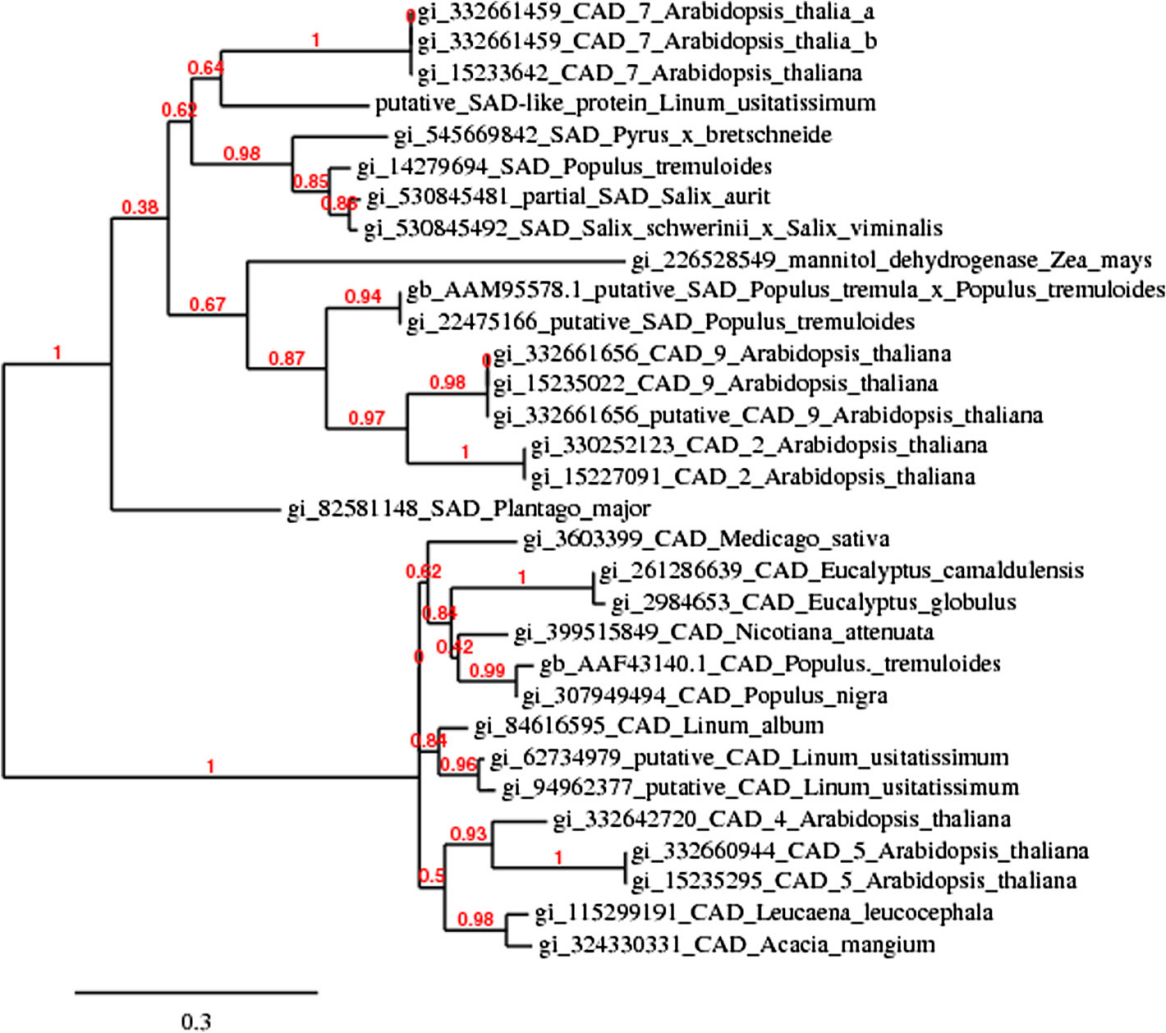
**The phylogenetic analysis of 29 selected CAD/SAD putative or verified proteins from angiosperm and gymnosperm and two investigated proteins: putative CAD and SAD from *Linum usitatissimum*.** Numbers above branches refer to branch support values.

CAD is the final enzyme on the monolignol biosynthesis pathway. So far, only one whole *CAD* transcript from *Linum usitatissimum* L. is available in the database [GenBank: DQ487210.1] and is referred in the manuscript as flax CAD. Furthermore, two partial sequences of *CAD* were identified in flax [GenBank: EU684538.1 and AY837831.1]. NCBI Blast alignment of flax *CAD* showed the highest similarity (77-76% of identity) to a number of *CAD* sequences from genus *Populus* [GenBank: XM_002313839.2, AF217957.1, AY479972.1, EU760897.1, Z19568.1] and *Eucalyptus* [GenBank: GQ916948.1]. Figure 2 presents biochemical relationship between flax CAD protein [GenBank: 94962377] and a set of characterized CAD proteins from wide range of plants.

Although CAD is established as a major enzyme that catalyzes all three monolignols biosynthesis, SAD is also reported to take some part in lignin biosynthesis [11-14]. Thus, when *CAD* was down-regulated there was a suspicion that its function might be overtaken by an enzyme with similar activity. *SAD* gene mRNA. [GenBank: AF273256] from *Populus tremuloides* was a probe to screen *Linum usitatissimum L.* genome [GenBank: AFSQ00000000.1]. As a result a homologues (72% of identity) 648 nt sequence was found. It showed no homology with the flax CAD and high similarity with the number of sinnapyl/cinnamyl-like alcohol dehydrogenases from various plants. Moreover, the putative SAD sequence was translated into amino acid sequence and phylogenetic analysis was carried out (Figure 2). It turned out that the protein in question is closely related to CAD7 protein form *Arabidopsis thaliana* and SAD from *Populus tremuloides* [GenBank: AAK58693.1], *Salix aurit* [GenBank: AGT52199.1], *Pyrus bretschneide* [GenBank: AGW45368.1] and some others. Moreover, PHYTOZYME BLAST alignment classified the protein as an alcohol dehydrogenase, class V with oxidoreductase activity and zinc ion binding domain.

The genes expression analysis with semi-quantitative PCR was carried out on *in vitro* cultures from *CAD*-reduced flax. It appeared that *CAD* silencing led to reduction in *CAD* gene activity by 40% and 55% for CAD27 and CAD33 respectively. Surprisingly, it was compensated by reasonable over-expression of *SAD* gene, to 220% and 365% of the control (100%) respectively.

Further genetic analysis is needed on the flax plants to identify CAD isoforms in flax, as well as other research to confirm SAD enzyme activity in flax. Moreover, other transgenic respective events in modified CAD flax plants should be investigated as they might contribute to the strength of CAD silencing and plant growth and development.

### The phenotype of *CAD*-silenced plants from the field trial

Full analysis of modified flax plants with reduced lignin level (CAD plants) [50] requires obtaining flax crop, which means the need of a field trial. Owing to the fact that cultivating transgenic plants in a field is an experiment itself, a plant phenotype analysis was done.

For two chosen lines, CAD27 and CAD33, four parameters taken into account were plant height, number of seeds in the capsule, seeds weight and straw yield expressed as fibre weight possessed from the straw per straw weight (Table 1).

**Table 1:** The phenotype parameters measured for plants from two transgenic lines and the control plants (wild-type)

**Plant height**		
[cm]	2009	2010
WT	83,1	68,7
CAD 27	87,4	66,7
CAD 33	70,7 **	53,1 **
**Seed weight**		
[mg]	2009	2010
WT	5,46	4,91
CAD 27	5,49	4,82
CAD 33	4,74 **	4,11 **
**Number of seeds in the capsule**		
	2009	2010
WT	6,52	6,22
CAD 27	7,22	6,15
CAD 33	6,90 **	4,34 **

Plants were grown in the field and collected annually in years 2009–2010. **- p < 0.0001.

The results obtained in the first year showed that first three factors did not significantly change for line CAD27 as compared to the control plants, whereas line 33 noticed measurable decrease, about 15-20% for all three parameters. In the next vegetation season the results agreed (Table 1). Although the numbers were different, as they depend on many environmental factors, the tendency was the same.

Another measured parameter was straw yield, expressed as mass percentage of fibre in the straw. The results showed that both lines, CAD27 and CAD33, contain less fibre than non-modified plants, 88% and 80% of the control (100%) respectively.

This indicates that these two lines responded differentially to the introduced modification and expressed different phenotype. Additionally, the modification of lignin synthesis pathway resulted in lower straw yield. However, for line CAD33 measured parameters slightly declined, it did not disturb plants growth and development *in vivo*.

### The resistance against pathogen infection

It is well established that lignin participates, as a mechanical barrier, in plant defense against pathogen infection [2]. To verify how repressing CAD affects plants resistance against flax major pathogen fungi from genus *Fusarium* seeds obtained in the field trial were subjected the infection test using the mycelium method. It appeared that both tested transgenic lines, CAD27 and CAD33, were more vulnerable to the *F. oxysporum* infection by 36% and 17% respectively as compared to the control (100%). However, susceptibility to the infection did not exceed 140% of the control in the laboratory test, and in the field trial effect of the *F. oxysporum* infection was not observed.

On the contrary, talking about *F. culmorum* resistance, line CAD27 showed 39% decrease in the number of infected seedlings, whereas line CAD33 was about 22% more vulnerable to the pathogen.

### Retting efficiency

Lignin is known to be the mechanical barrier for the microorganism in the pathogen attack, as well as a stumbling block for the pectin-degrading enzymes during retting. Therefore, retting efficiency test was carried out by means of monitoring pectin level in the retted straw. The representative samples were taken from the straw retted in the field with dew method in the zero, eighth, fourteenth, seventeenth and the last, twentieth day of retting. Then, dried and grounded to powder, samples were examined according to the standard procedure of the total pectin analysis.

**Figure 3:**
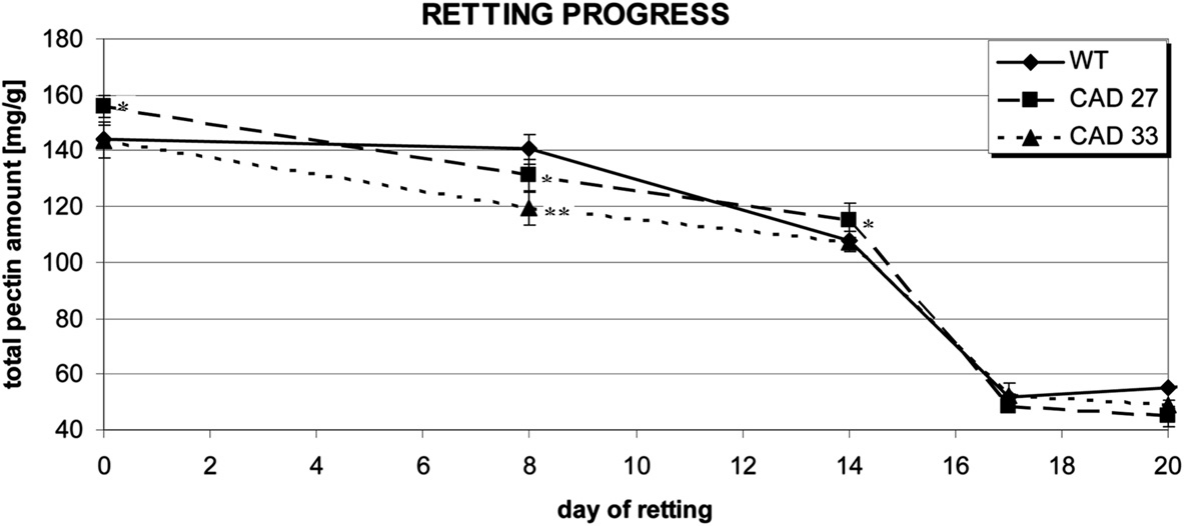
**The total amount of pectin level measured in the flax straw during dew retting.** * - p < 0.006; ** - p < 0.00001.

The research revealed that mature stalks from line CAD27 have slightly higher, for about 8%, total level of pectin, whereas CAD33 and the control plants had the same pectin amount (Figure 3). The most remarkable results were obtained after eighth day, when strong decrease in the pectin level was observed for both transgenic lines and its amount was visibly lower that the control one. Moreover, a graph pattern for the pectin level in transgenic lines was nearly identical, while the control graph had the alternative shape. For CAD-silenced straw the graph showed clear linear fall in first 14 days and then the strongest drop was observed between 14th and 17th day. On the contrary, the graph for the wild type straw was quite flat in the first eight day, and then between 8th and 17th day the fall was observed.

It seems that even if the overall retting time was not shortened, CAD straw was colonized by microorganisms much quicker, and the pectin degradation enzymes acted effectively from the very beginning of the retting. Furthermore, the process of retting in the transgenic straw was more uniform, which might contribute to an improvement in the fibre quality.

**Figure 4:**
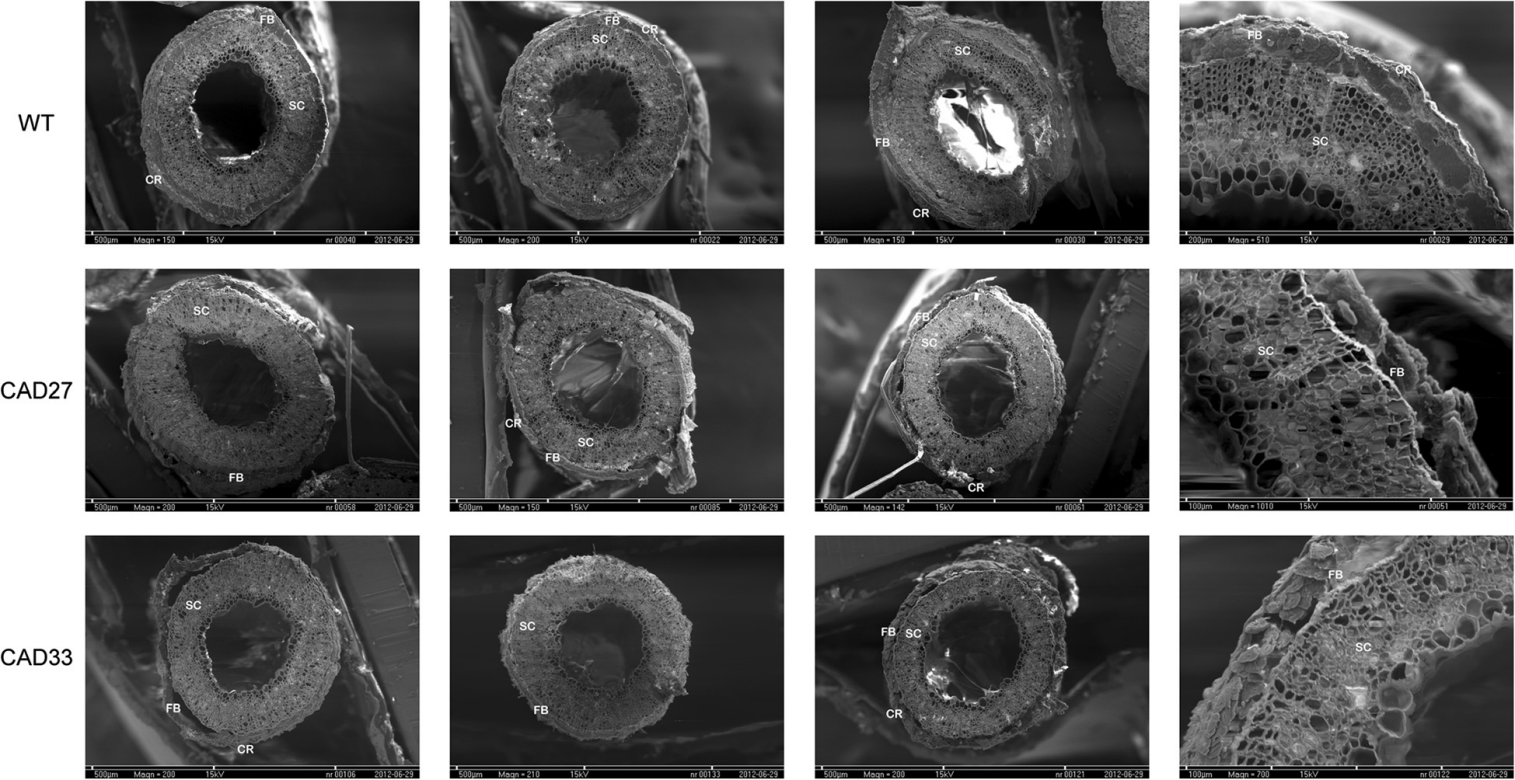
**Scanning electron microscopy for retted flax stalks.** These are representatives of 5 specimens (from 5 different stalks) prepared for each line and the control plants. It appeared that although total pectin level was nearly identical for both, transgenic and control plants, in stalks of the first retting is more advanced. SC – stalk core, FB – fibre bundles, CR – cortex.

To confirm the differences in retting efficiency and quality SEM was performed on retted straw. Stalks from the very last day of retting were dried and probed under SEM microscope, the representative specimens are presented in Figure 4. For both transgenic lines it is clearly visible that cortex is discrete heavily whereas in the control the process only started to happen. Even more, fibre bundles in low-lignin samples were separated from the core in nearly 100%. These could not be seen in the control samples.

To sum up, all mentioned factors indicate that indeed silencing *CAD* gene resulted in an improved retting efficiency.

### Biochemical analysis of flax fibre from plants with disturbed lignin biosynthesis pathway

**Figure 5:**
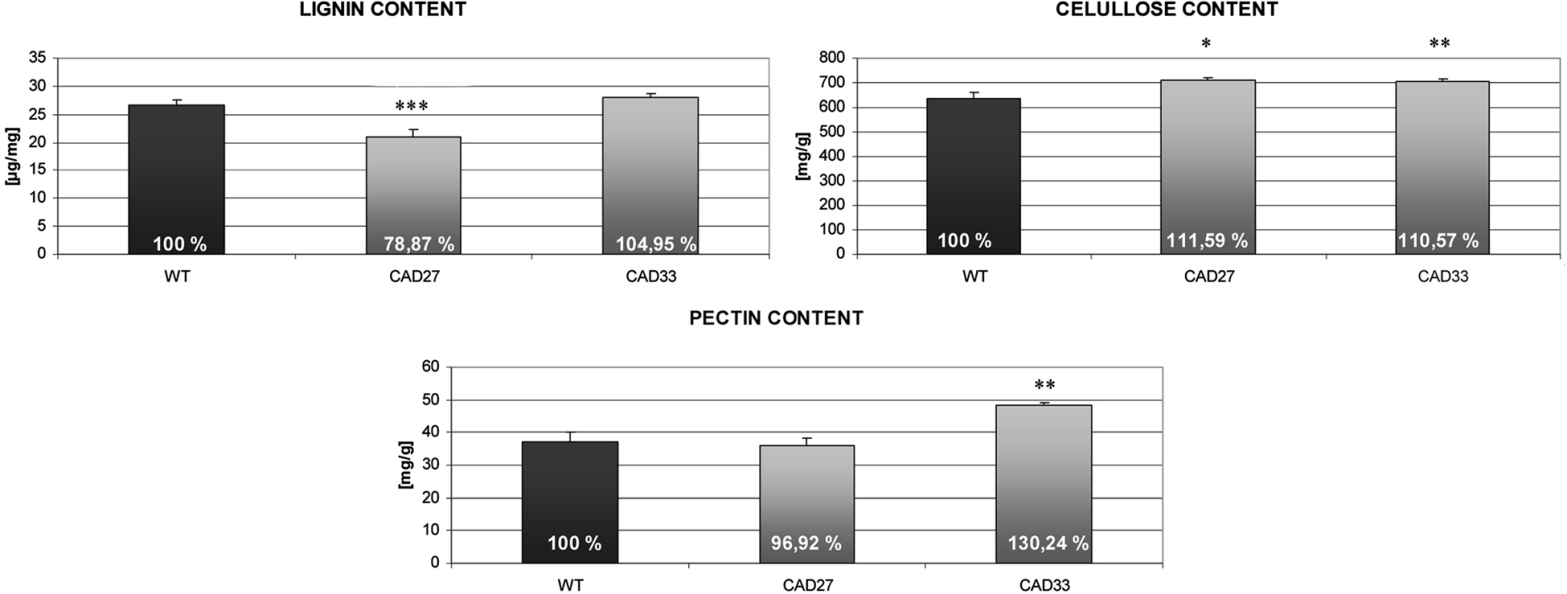
**The amount of major components in flax fibre.** * - p < 0.05; ** - p < 0.008; *** - p < 0.0003.

Since the modified gene, *CAD*, is one of the key enzymes in the lignin biosynthesis pathway, primarily the lignin amount was investigated in fibre possessed from the field trial. The results revealed that in the case of fibre from CAD27 line, lignin level significantly decreased, nearly 20% fall was observed (Figure 5). On the contrary, CAD33 fibre showed no measurable difference in the lignin level as compared to the control plants. However, taking into account the lignin/cellulose ratio it was reduced in both transgenic lines. In particular, CAD27 noticed 30% reduction in the lignin to cellulose relation, whereas CAD33 showed only 5% decrease.

**Figure 6:**
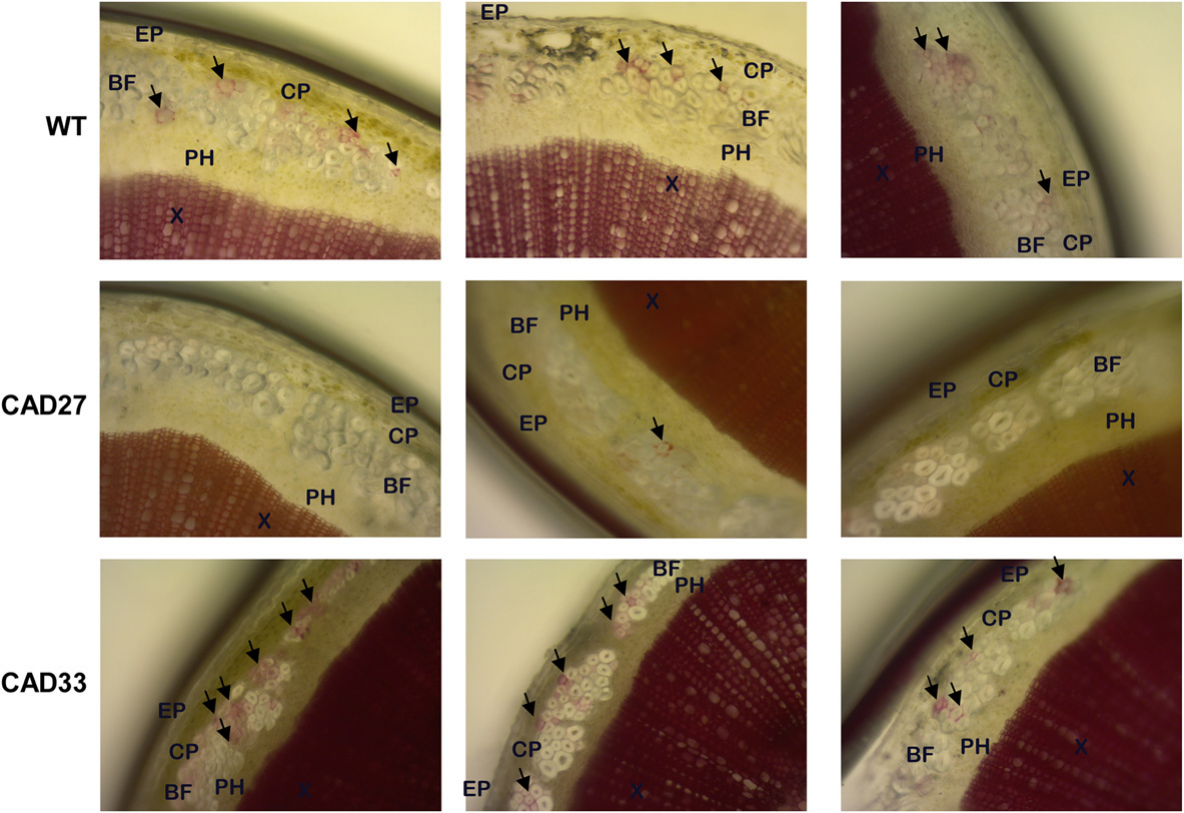
**Lignin staining in a basal-section of the flax stalks grown in a field.** Arrows point at places where lignification of the fibre cell wall has just started to happen. These are three representatives of 20 specimens prepared for each line and the control plants (wt). Magnificent: x40. EP–epidermis, CP–cortical parenchyma, BF–bast fibre cells, PH–phloem, X–xylem.

To confirm obtained results, we examined phluoroglucinol-HCl stained cross sections of 10 weeks old plants grown in the field (Figure 6). The samples were taken just after blossoming, when the lignification of the cell wall in fibre starts to happen [40]. This allowed us to see putative difference on the early stage of the fibre’s cell wall development. The results showed that in CAD27 line, lignification was delayed and/or reduced; only singular points where the process developed might be seen. We suspect that this is one of the putative effects of the introduced modification. In addition, lignin staining showed that xylem and phloem cells had normal morphology.

For the rest of the main components in the fibre cell wall, the cellulose level also has changed, for both transgenic lines 10% increase was observed (Figure 5). This was followed by changes in the pectin amount. It appeared that while CAD27 fibre showed no telling changes, CAD33 fibre had 30% more pectin than the control.

**Figure 7:**
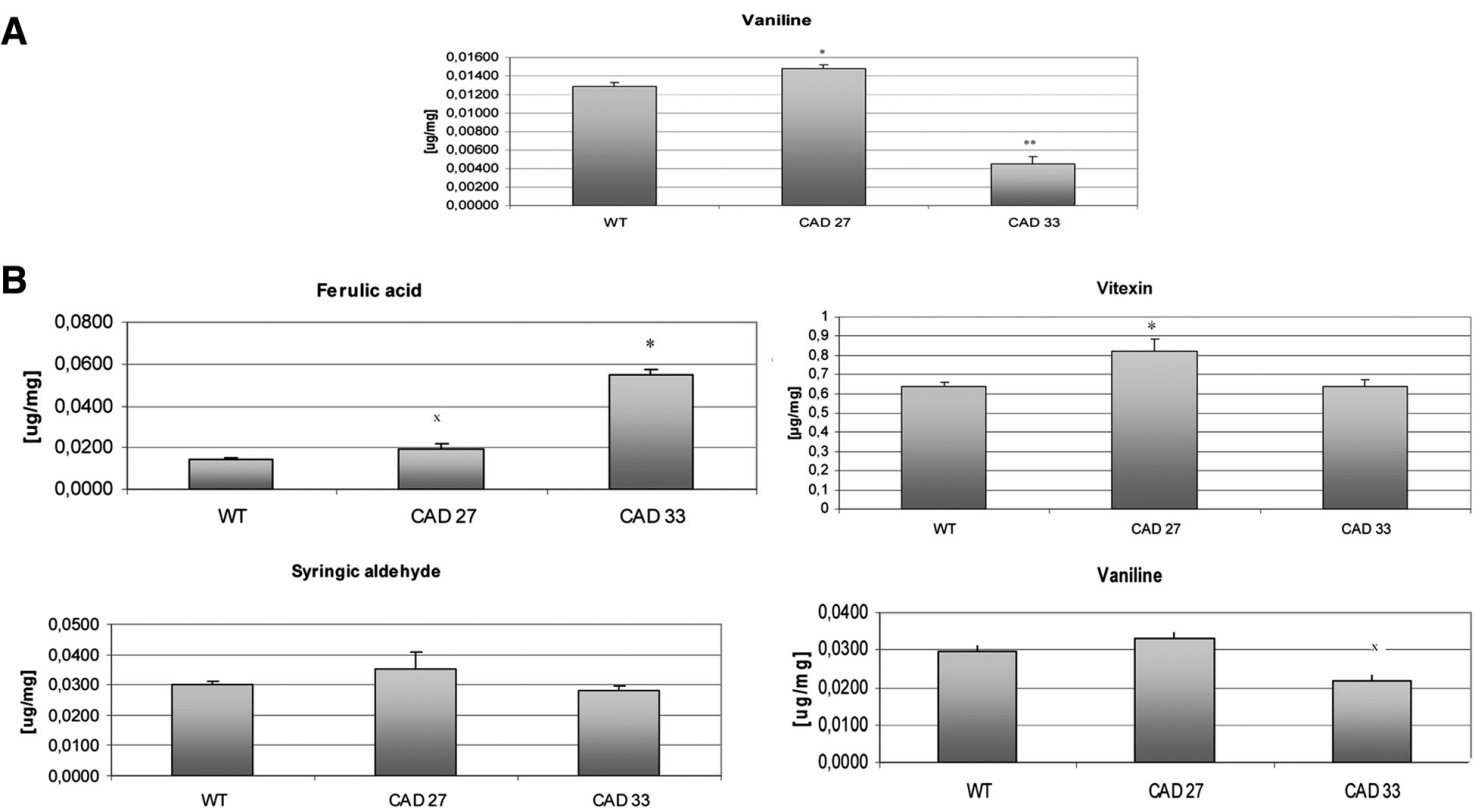
**Determined with UPLC content of phenylpropanoids linked to the fibre cell wall (A) by hydrogen bonds, (B): ester-bounded.** x – 0.19 > p > 0.08; * - p < 0.02; ** - p < 0.00008.

The analysis of main components of the cell wall was followed by the investigation of metabolites from the phenylpropanoid pathway as part of it was disturbed. To achieve the task, two extractions were carried out. First, with methanol, was conducted to identify free components. The only detected phenolic was vanillin (Figure 7A), whose content was three fold lower in CAD33 fibre, whereas CAD27 fibre noticed slight increase when compared to the non-transgenic fibre.

To release metabolites ester-linked to the ligninocellulosic polymer, an alkali hydrolysis was conducted. Apart from vanillin, detected compounds were ferulic acid, syringaldehyde and vitexin (Figure 7B). The rest of the components were in traces amount, not possible to identify.

The fibre from CAD27 line was characterized with slight increase in the content of vanillin, ferulic acid and sirigaldehyde. Vitexin, an apigenin glicoside classified as flavanone, was also detected in the fibre extract. Its amount was about 30% higher in the CAD27 fibre as compared to the control fibre.

CAD33 fibre showed high amount of ferulic acid, nearly three times more that in the control fibre, vanillin amount was 30% smaller than in non-transformed fibres, whereas the amount of siringaldehyde and vitexin did not significantly changed.

### Vibrational data for fibre

There is evidence that manipulation in lignin biosynthesis pathway results not only in lower lignin level but also changes its structure and thus rearrange of the polymers. To picture the chemical character of the cell wall polymers and its linkages the infra-red spectroscopy analysis was carried out [51-56].

**Figure 8:**
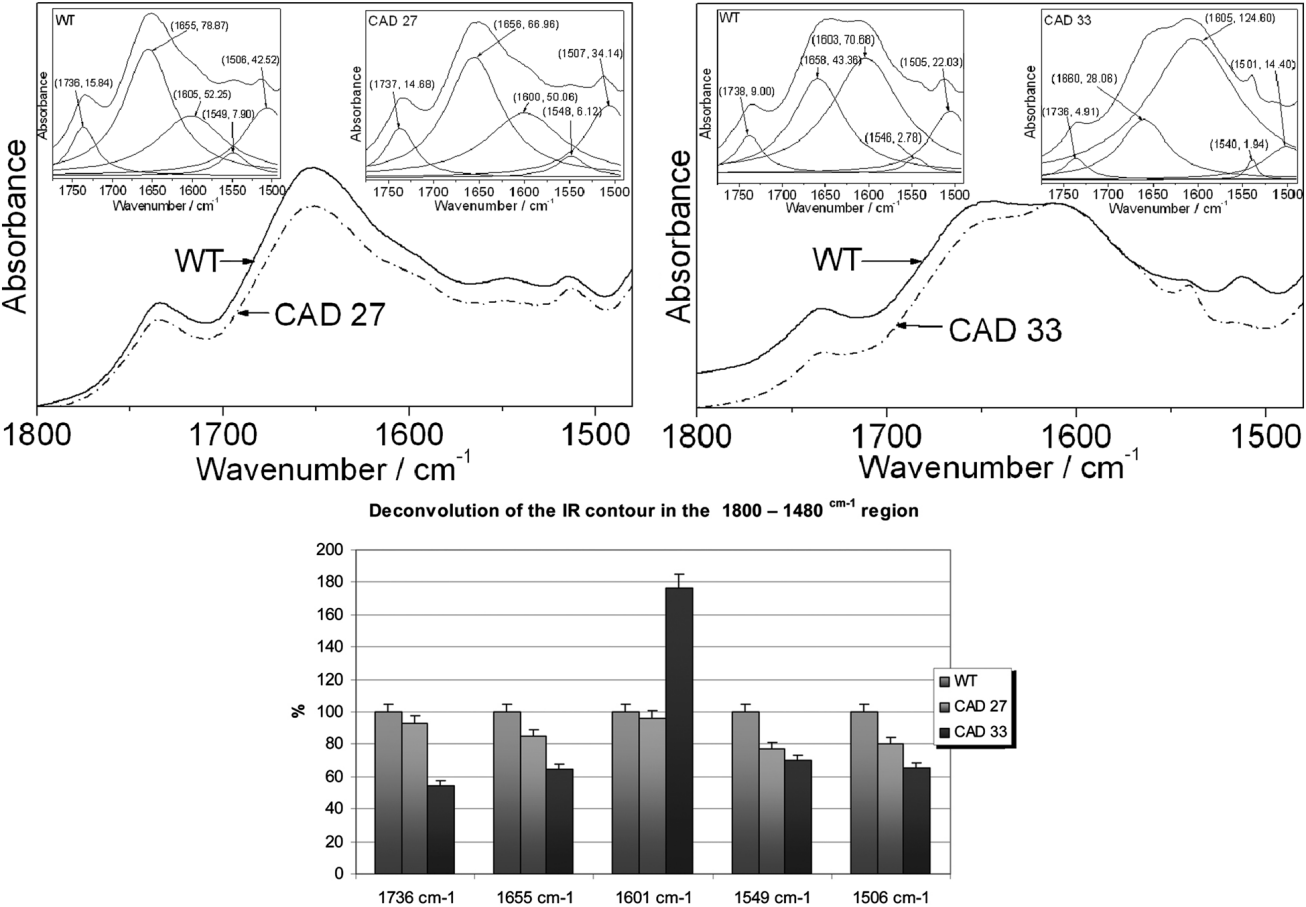
**IR spectra of the studied samples in the 1800–1480 cm**^**-1**^** range.** The energetic positions and integral intensities of the Lorenzian components are given in the parentheses.

The IR bands in the 1800 – 1480 cm^-1^ region were used for identification of the changes in the lignin and pectin content. Figure 8 shows the IR spectra together with the Lorenzian distributions of contour observed in this region, which are deconvoluted into five Lorenzian components. The first three components at about 1736 cm^-1^, 1655 cm^-1^ and 1605 cm^-1^ correspond to the pectin groups, ν_as_(COO) vibrations of unconjugated and conjugated carboxyl group of pectin [54].

Although the relative intensity of these bands differentiate in numbers they confirm that in CAD33 fibres there is much higher pectin amount, which indicate the strong increase in the third band. The integral intensities for all three bands are slightly lower for CAD27 line, which is in the agreement with biochemical analysis.

Bands observed at 1500 and 1545 cm^-1^ were described as pectin and lignin - related absorbances [57,58]. The integral intensities of the analyzed bands at about 1548 and 1506 cm^-1^ decrease in the case of lines CAD27 and CAD33. It suggests that there is lower amount of the lignin in both transgenic lines.

**Figure 9:**
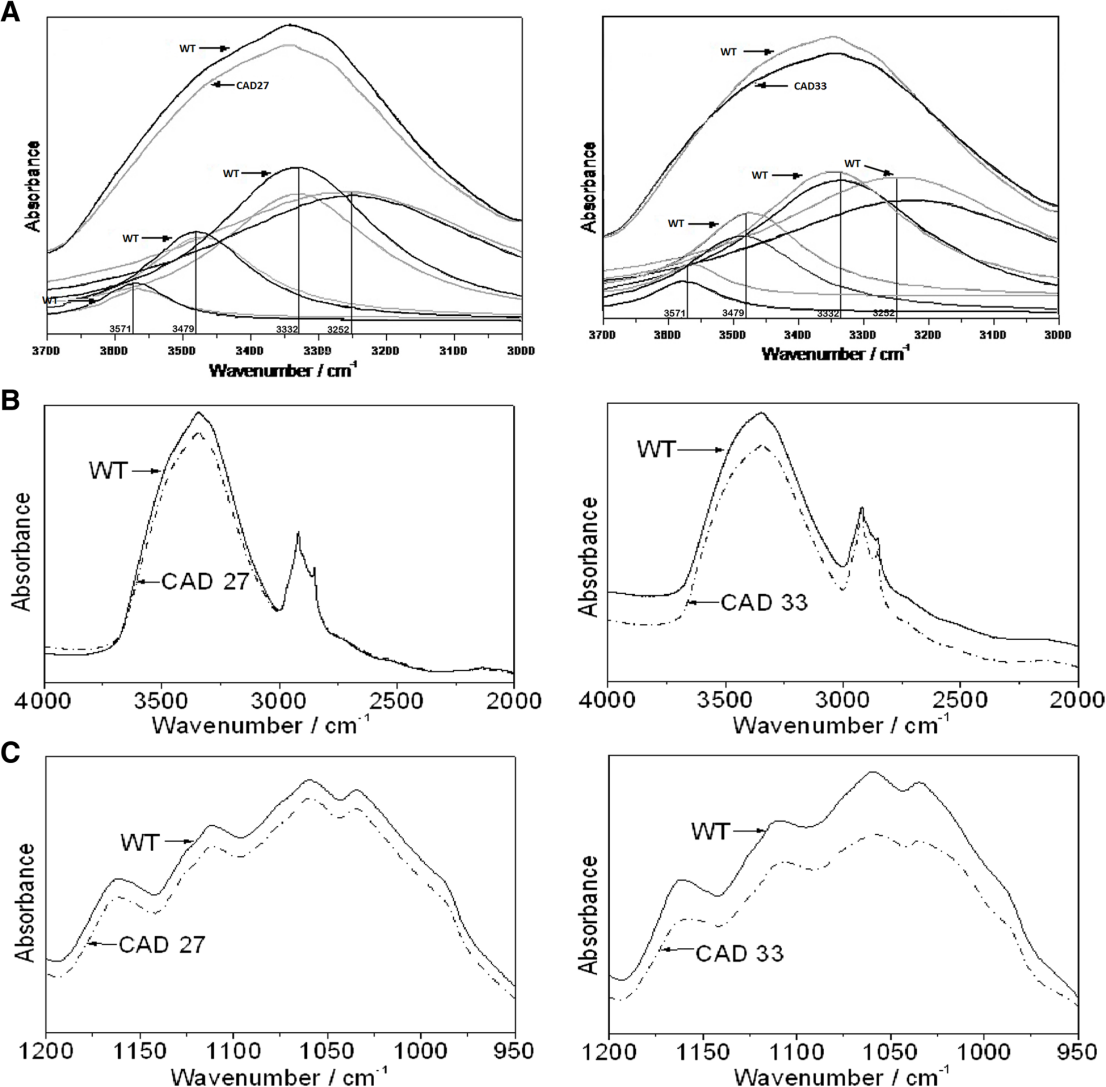
**IR spectra of the studied samples. (A)** Deconvolution of the IR contour in the 3700–3000 cm^-1^ region. Lorenzian distributions of the contour are deconvoluted into four components: 3571, 3479, 3332, 3251 cm^-1^. The IR contours in the region **(B)**: 4000–2000 and **(C)**: 1200–950 cm^-1^ of the control (wild type) and modified flax fibres.

The broad absorption band at 3400 cm^-1^ corresponds to the stretching -(OH) mode of the free hydroxyl groups and those involved in the intra- and inter-molecular hydrogen bonds (HB). In particular, the Lorentzian distributions of this contour can be deconvoluted into four components: 3571, 3479, 3332, and 3251 cm^-1^ (Figure 9A). First three of them correspond to the intramolecular HBs in the cellulose molecule whereas the fourth component corresponds to intermolecular HBs [59].

The intensity of the contour expanded from 3000 to 3600 cm^-1^ clearly decreases for transgenic flax fibres (Figure 9B). The shape of this band is nearly the same for all the studied samples (i.e. the control and transgenic fibres), but the bands differ in terms of their absorption intensity. Studying the absorbance for the Lorenzian components (Figure 9A) it appeared that first three of them clearly decrease for both transgenic lines, and stronger decline is observed for CAD33 line. On the contrary, band 3251 cm^-1^ noticed slight increase in the intensity for CAD27, while CAD33 showed quite strong decrease.

Putatively, it might be concluded that cellulose molecules in fibre from line 33 contain less HBs, both intra- and intermolecular, whereas in CAD27 there are less HBs within cellulose molecule, but there are more of them between cellulose molecules themselves, or between cellulose and other molecules. Additionally, the decrease in the intensity of the contour at about 1050 cm^-1^ for the transgenic flax suggests that the cellulose polymers are shorter in genetically modified plants because they contain less C-O-C bridges (Figure 9C).

Summarizing, the FT-IR analysis allowed to verify lignin and pectin level in the analyzed fibres and gave an insight into cellulose structure and interactions within the cell wall polymer. It appeared that for both transgenic lines cellulose chains are putatively shorter and contain less HBs within their molecules. In case of intermolecular HBs, their number increase slightly for CAD27 samples, whereas reverse effect is observed for CAD33 samples.

### Mechanical properties

The aim of the genetic modification was to inhibit lignin biosynthesis in flax plants and by extension reduce their level to possible enhance mechanical performance of retted fibres. Tensile tests conducted on transgenic fibres from CAD27 and CAD33 lines showed that, indeed, an increase in the strength of the fibres was observed, relatively to the control plants by about 26% and 17% respectively (from 391 MPa for the control, to 492 MPa for the CAD27 and 456 MPa for CAD33, for both lines p < 0.007). In turn, the tensile stiffness, measured as the Young’s modulus, increased in CAD27 by about 24% and in CAD33 by about 8% (from 129 GPa for the control, to 159 GPa for the CAD27, p < 0.0006 and 139 GPa for CAD33). All these changes in the mechanical parameters corresponded to the extent of lignin level, and also cellulose, together with the lignin to cellulose ratio reduction, being more pronounced in the former line than in the latter.

## Discussion

### CAD and putative SAD proteins in flax are phylogenetic distinct

Phylogenetic analysis and comparison of its results with phylogenetic analysis of CAD family strongly suggest that flax CAD protein [GenBank: 94962377] belongs to the Class I - „bona fide” of CAD family (Figure 2) referring to both Guo et al. and Barakat et al. classification [8,13]. Although these two classifications have some differences, in general Class I consist of species from monocots, eudicots, and gymnosperms and includes two *Arabidopsis* CAD proteins (*AtCAD5* and *AtCAD4*) [60] associated with lignin biosynthesis. Putative flax SAD protein belongs to the Class II-A-SAD referring to the Guo et al. classification and to Class II by Barakat et al. His team concluded that CADs from Class I in *Populus* are responsible for xylem lignin biosynthesis whereas Class II and III proteins show the highest activity under stress conditions. Putatively, in flax, CAD is also responsible for lignin synthesis in conducting tissues (xylem, phloem), whereas SAD plays supporting role [8,11-14,61].

### *CAD* down-regulation did not affect plant growth and development *in vivo*

In this study we correlated introduced modification with its effect on phenotype, composition and properties of transgenic flax *in vivo*. Since lignin is known to support plant stalk, it is necessary to evaluate how lowering lignin level affect plant development. Two lines chosen to the field trial showed different growth.

There are reports that lignin reduction by silencing genes from lignin biosynthesis pathway (including CAD) causes stunned growth in *A. thaliana* and *Pinus radiate*[15-21,62,63]. However, it was reported recently that *Nicotiana attenuata* plants with silenced *CAD* gene, had robust phenotype when grown in a greenhouse, but showed normal phenotype when planted in a field [19]. It was found that down-regulation had not regressed, only various compounds where inbuilt in lignin structure to ensure up-right orientation of the stalk. Another research showed that suppression of lignin biosynthesis pathway genes in *Brassica napus* caused reduced lignin level, but the modifications did not disturb plant growth [64]. Similarly, reducing lignin level by silencing genes involved in lignin biosynthesis, CAD among them, in flax, maize and switchgrass did not cause alterations to plant growth [3,23,65].

Most probably this is what happened in case of CAD27 and CAD33. Indeed, flax plants with the strongest *CAD* repression showed stunned growth and did not survived, whereas CAD27 and CAD33 do not have the strongest gene silencing [50], and moderate lignin reduction does not affect plant growth and development. The field trial did not reveal serious deterioration of the crop in comparison to the non-modified control plants, although it cannot be disregarded that CAD33 had slightly worse growth parameters than CAD27.

Moreover, lignin staining (carried out on plants *in vivo*) had not shown any morphological changes in the shape or size of the conducting cells. There is evidence that CAD down-regulation is correlated with collapsed vessels and red-brown colour of the xylem cell [15,64,66]. However, it is also stated that to some extension, reducing lignin level did not cause any morphological changes [2,15] and this is what we observed, no collapsed vessels or red-brown colour of conducting tissues were observed.

Straw yield was fourth measured parameter, as it is one of the most important economic factors. Although plant growth in both lines was not significantly disturbed, reduction in straw yield was observed. Reducing lignin level is known to have negative impact on crop yield [4]. Another modification, introducing additional polymer to the fibre cell wall structure also resulted in lowering straw yield [67], whereas an over-expression of potato’s β-1,3-glucanse in flax had no impact on straw yield as compared to the control [68].

### Silencing *CAD* gene results in changed resistance to pathogen infection

Indeed, in case of *Fusarium oxysporum*, the main flax pathogen [69] being responsible for *Fusarium* wilt, both transgenic lines showed increased vulnerability to the infection. This fungi attacks plant through the water conducting vessels, therefore reducing lignin level and/or changing its structure (improving their digestibility) is a clear reason for the obtained results.

It is well established that lignin acts as a physical barrier against initial ingress; however, there is evidence that in some cases plants with reduced lignin level have improved resistance to certain pathogens [70]. For instance, sorghum with lowered lignin amount had increased resistance to some *Fusarium* spp. strains [71].

Since *F. oxysporum* and *F. culmorum* have different infection mechanism, and two flax CAD lines showed different metabolism it is possible that transgenic types have different level of resistance against those pathogens. These also might contribute to the diversity in the CAD27 line resistance against *Fusarium* fungi. Furthermore, *F. culmorum* is not a pathogen specific only for flax; it is generally a plant pathogen. The hypothesis requires further careful investigation on plants metabolism and the gene expression pattern.

Low-lignin plants are of great interest for biotechnologies due to biofuel production form plant biomass [48]. At the moment a various plant species are engineered to have lower lignin level, and thus filed trials are required to determine characteristic and phenotype of newly obtained plants [70]. Indeed, for flax *CAD*-silenced lines increased susceptibility to the pathogen infection and reduced straw yield are flaws when thinking about potential application. However, the experimental field trial did not reveal any serious deviation in growth as compared to the control. So, all in all, more field trials on a large scale are essential to give an ultimate decision on agricultural and industrial application of our lignin-low flax types.

### Repressing *CAD* resulted in changes of the composition and structure of flax fibre cell wall

The crop from plants with silenced *CAD* gene was expected to show decline in lignin level. Indeed, in fibre from line 27 strong decrease was observed, whereas line 33 noticed no significant changes in total lignin amount (Figure 5). However, FT-IR vibrations characteristic for lignin showed clear fall in the absorbance intensity (Figure 8), which may indicate changes in the number of specific bonds and thus changes in lignin structure and/or composition. Moreover, SEM and retting efficiency test showed for both transgenic lines increased susceptibility to the polysaccharides degradation by microorganisms. Additionally, *CAD* and *SAD* genes expression analysis revealed that CAD repression was compensated by reasonable over-expression of *SAD* gene, particularly for CAD33.

These allowed us to draw a conclusion that both lines have altered lignin composition and structure, whether it was connected with change in total lignin amount (CAD27) or not (CAD33). The gene expression evidence might also be a molecular explanation to the fact that both lines have different composition and properties. Moreover, the number of studies on lignin reduction by repressing genes from the lignin biosynthesis pathway (*CAD* among them) showed that not always the modification results in reducing lignin level but sometimes only the lignin composition and chemical interactions are changed for instance by incorporation of the hydroxycinnamic acids into lignin structure [15-21].

The remaining sugar components, like cellulose and pectin, also changed their amounts in the modified CAD lines. For both lines the accumulation of cellulose was observed, and in case of CAD33 also pectin increase. It was not an unexpected result, as it was reported that modification in the lignin biosynthesis pathway may cause increase in cellulose level. In maize with *CAD* repression the level of CAD activity varied between plant tissues, which resulted in different effects of the modification depending upon the tissue [65]. In stalks of transgenic maize, the total lignin content remained unchanged whereas accumulation of cellulose and arabinoxylans occurred. In contrast, cell walls of CAD-RNAi midribs present a reduction in the total lignin content and in the cell wall polysaccharides amount. For other maize mutants with altered lignin content similar effect occurred, pectic polysaccharides were accumulated [72]. Also in transgenic aspen with silenced 4-coumarate-CoA ligase (4CL) strong decrease in lignin level (up to 45%) was accompanied by about 15% increase of cellulose level [73]. We speculate that *CAD* down-regulation resulted in relaxation of the lignin structure and/or reduction in lignin amount, which was demonstrated in the FT-IR results (Figure 8) and biochemical analysis (Figure 5), and these changes were somehow correlated with the increase in the cellulose and/or pectin amount to support integrity of the cell wall itself. Tightening bast fibre cells by accumulating pectin/cellulose is a way to maintain an up-right orientation of the plant stalk, its mechanical resistance and by extension proper growth and development. Although the molecular background of this compensation remains unclear, there is wide evidence that such compensatory mechanism is present in plants [74-76].

### *CAD* down-regulation did not lead to the accumulation of lignin precursors

Phenylpropanoid compounds are wide and important group of secondary metabolites, which play various roles in plant growth, development and plant defense against pathogens. They are also plants’ pigments [77-79]. However, for fibre, they are of great importance because they assure biological activity which distinguish flax fibre among other natural fibres.

For CAD27 fibre there is an increase in the flavone (vitexin) content, but otherwise accumulation neither of lignin precursors, nor compounds of the other branches of the phenylpropanoid pathway occurs. Surprisingly, in line 33, total vanillin level decreased heavily, while strong rise in ferulic acid content was observed, which was accompanied by no changes in the sringaldehyde amount. In plants vanillin could by synthesized in the phenylpropanoid pathway, being biotransformed from ferulic acid [80]. It seems that this conversion was blocked, but molecular background of this fact remains unclear.

Putatively, to support lignin integrity 4-coumarylo-CoA instead of being substrate for anthocyans or flavanone synthesis was shifted to the lignin biosynthesis pathway. Further, monolignols precursors were inbuilt in lignin structure and this is a reason why we could not see their accumulation. Such mechanism, reinforcing lignin with hydroxycinnamic acids when CAD is down-regulated is described widely in literature and was described in previous paragraph [15,16,18-21].

### Vibrational data confirmed chemical analysis and proves changes in conformation and structure of chemical bonds in the cell wall

Lignin, apart from cellulose, hemicelluloses and pectin, is the next polymeric composite that appears in the plants. FT-IR spectroscopy is a useful technique for determination of the lignin content in pulp and plant. The vibrational properties of lignin derived from different plans have been defined in details and widely used for their characterization [52-56].

The regular changes in the IR intensity of the 3400 cm^-1^ band, corresponding to the hydroxyl groups in phenolic and aliphatic units, are observed for the control sample and genetically modified flax fibres, CAD27 and CAD33. The overall intensity of all the bands for the investigated samples follows the direction I_WT_ > I_CAD27_ and I_WT*_ > I_CAD33_ (Figure 9B). The changes in the intensity of the 3400 cm^-1^ band components for the control and transgenic fibres probably resulted from different conformations of the intramolecular and intermolecular HB (O-H^…^O) of the glucopyranose system.

Such changes are expected when different rotary isomers appear in the skeleton of the cellulose since they differ in the strength and orientation of the HB. This leads to the disordered arrangement of the pyranoid rings in the cellulose polymers in the genetically modified flax.

The contour from the 950 – 1200 cm^-1^ range corresponds to the ν_as_(C-O-C) vibrations. This result confirms that the amount of the HB is different in the natural and genetically modified flax fibres. The intensity decrease of the contour at about 1050 cm^-1^ for the transgenic flax suggests that the cellulose polymers are shorter in genetically modified plants because they contain less C-O-C bridges.

### Manipulating lignin level in flax plant led to improve fibre mechanical properties

Posttranscriptional silencing of CAD genes affects lignin biosynthesis and cell wall chemical composition, therefore significant modifications in the mechanical performance of flax fibres occurred. The mechanical effect of the genetic modifications depended, however, on the line of transgenic plants and was related to changes in the chemical cell wall composition. Particular improvement in the tensile strength and tensile stiffness was observed in CAD27 fibre that apparently corresponds to the increase in the fibre content of cellulose, the main load-bearing structural component of the composite cell wall material [81], and relative decrease in the lignin and pectin content, as compared to the control (Figure 5). Additionally, it might also correlate with lignin/cellulose ratio, as CAD27 fibre has significant fall (around 30% comparing with the control) whereas for CAD33 fibre only 5% was noticed. The mechanical properties of tested fibres were characterized by the maximum force recorded during stretching performed at relatively high extension rate, referred to the original surface area of the cell wall material. Therefore, the increase in the strength observed for the transgenic plants reflected the improvement in the material properties of the cell wall and/or in the interfacial adhesive interactions between the neighboring elementary fibres. The differences in these material properties may also be explained by the different abundance of defects accumulated during growth and/or raw fibre processing [82].

## Conclusion

It appeared that the two examined kinds of fibre, from transgenic lines CAD27 and CAD33, responded differently to the introduced modification. This might indicate that changes in the phenotype of the low-lignin plants are not only connected with the *CAD* silencing, but also with the fact of the genetic modification itself (the construct location in the genome), and efficiency of the modification (the number of copies inserted into flax genome). To conclude that all observed changes are trustworthy and correlated exclusively to CAD repression, further analysis of the modified plants’ genome is necessary.

However, flax plants from field trial with the modification targeted directly to block lignin biosynthesis were characterized not only by reduced lignin level (in one transgenic line), but also by altered amount and organization of the other cell wall components. This is a contribution to the gathering knowledge about compensatory mechanism existing in plants. Further careful evaluation on the molecular basis of the observed alterations is under investigation.

In particular, CAD down-regulation in flax:

•does not disturb at all or has only slight effect on plant’s growth and development in vivo, however it reduces straw yield in flax;

•results in more uniform and efficient retting;

•has different effects on plants’ resistance; increases vulnerability to *F. oxysporum* attack, but induces resistance to *F. culmorum* (one transgenic line);

•affects lignin composition and structure in the cell wall of flax fibre;

•changes the cellulose structure and organization (cellulose chains are putatively shorter and contain less HBs within their molecules; however CAD27 and CAD33 differentiate in the number of intermolecular HBs);

•activates compensatory mechanism in the fibre cell wall, rearrangement in the fibre cell wall composition occurred;

•causes improved fiber’s tensile strength, but it does not reduce its stiffness.

Finally, this is one of the first studies on the crop from the low-lignin plants cultured in the field trail. It demonstrates that such plants may have normal phenotype and be successfully cultivated in a field.

## Methods

### Phylogenetic analysis

All nucleotide and protein alignments were carried out using NCBI BLAST tool (blast.ncbi.nlm.nih.gov), nucleotide sequences were translated into amino acids using PHYTOZOME BLAST tool (http://www.phytozome.net) and phylogenetic analysis was done using TreeDyn software (via http://www.phylogeny.fr) [83-85].

### Gene expression analysis with semi-quantitative reverse transcriptase PCR

The total RNA was isolated from 4 weeks old plants from tissue culture using a TRIzol® reagent (Ambion®) following manufacturer’s protocol. The RNA integrity was verified with agarose gel electrophoresis 1.5% (w/v) containing 15% (v/v) formaldehyde. The RNA was cleaned from the DNA by DNaseI (Invitrogen) treatment. Then RNA served as a template for cDNA synthesis using a High Capacity cDNA Reverse Transcription Kit (Applied Biosystems). The sequences of primers that were used are presented in Table 2. Actin was used as a reference gene.

**Table 2:** The primer sequences, annealing temperatures (Ta) and number of cycles for semi-quantitative PCR reactions

**Gene**	**Primer’s name**	**The sequence of the primer**	**Ta [°C]**	**Cycles**	**Organism and GenBank number**
Actin	ACTforward	5’- CCGGTGTTATGGTTGGAAT-3’	61	**23**	Linum usitatissimum actin (Act1), [GenBank: AY857865.1]
	ACTreverse	5’- TGTAGAAAGTGTGATGCCAAA-3’	61	23	
CAD	CADforward	5’- GGAGCATGAAGGAAACAGAG-3’	62	**27**	Linum ustatissimum L. cinnamyl alcohol dehydrogenase (CAD), [GenBank: DQ487210.1]
	CADreverse	5’- CTACCTACGGAGGCTACT-3’	57	27	
SAD	SADforward	5’- CAACATCAACCACGAACCTA-3’	61.5	27	Linum usitatissimum scaffold726_21, [GenBank AFSQ01019765.1] on the basis of Populus tremuloides sinapyl alcohol dehydrogenase, [GenBank: AF273256]
	SADreverse	5’- GCTTGTCTAGCCCATAGAAC-3’	58.5	27	

AKT actin; CAD cinnamyl alcohol dehydrogenase; SAD sinapyl alcohol dehydrogenase.

The PCR conditions were 94°C for 3 min, and 27 cycles of 94°C for 25 s, 57°C for 30 s, and 72°C for 20 s and 72°C for 5 min. The number of cycles was determined depending on the transcription level to keep the reaction in the logarithmic stage (Table 2). The PCR products were visualized on 2% agarose gels with ethidium bromide under UV light. The levels of PCR products were measured by the densitometry analysis of the gel image system using the Bio 1D program. Densitometry was carried out using VisionWorksLS, UVP LLC software.

### Plant material

Flax plants with silenced *CAD* gene were generated as described by Wróbel-Kwiatkowska (2007) [50]. To avoid doubts that not all of *CAD* isoforms were silenced, a conservative fragment of 480 nt was chosen to be used in the transformation. It has over 90% of identity with a large number of *CAD* sequences from angiosperms. The two lines with significantly lowered CAD activity (as compared to the wild type plants) and phenotype not visually distinguishable from the control plants were chosen for further analysis. It was found out that the transgenic plants from *in vitro* cultures expressed nearly 40% decrease in lignin level measured with modified acetyl bromide method.

### Field growth and straw processing

Transgenic (1^st^ generation) and wild type plants were cultivated in the surrounding of Wroclaw in the years 2009–10. Harvesting was carried out after 4.5 months. On the randomly chosen bundle of 50 plants from each line and the control, the phenotype analysis was carried out by means of measuring plant height, stalk together with wisp, counting number of the capsules in the individual wisp and weighing at least 50 seeds for each sample.

Retting using dew method was conducted for twenty days. In this time the straw was turned over twice to ensure equal retting in the full straw volume. After drying, scutching and heckling the fibre was possessed.

### Determination of flax resistance to fungal pathogens

Seeds from transgenic plants and the control obtained in the field trail in 2010 were germianted on the MS medium. The 4-days old seedlings were transferred onto the medium (combined MS and PDA) with *Fusarium oxysporum* or *F. culmorum* strain and after 10 days the numbers of infected plants were determined as a percentage of the total plants used for the experiment.

### Lignin content

Total lignin content was measured with the modified 'acetyl-bromide’ method [86]. Briefly, dried and grounded into powder tissue samples were heated for 1 hour at 65°C with water and further filtrated through GF/A filters which was followed by washing several times with different organic solvents (in turn: ethanol, acetone, diethyl ether). Such prepared samples were dried for 12 hours and then, after adding 25% acetyl-bromide in acetic acid samples were incubated for 2 hours in 50°C and further dissolved in 10 ml of 2 N NaOH mixed with 12 ml acetic acid. After incubating for at least 12 hours at room temperature lignin content was measured spectrophotometrically at 280 nm. The results were given as an equivalent of coniferyl alcohol.

### Lignin staining

The experiment was carried out on green parts of the basal sections of the stalks grown in the field. From each line and the control 5 plants were taken to analysis.

Gently sliced into pieces, the cross-sections were transferred onto watchglass and stained with phluoroglucinol (Sigma-Aldrich)-HCl(12 N) solution for 5 minutes. Fresh specimens were probed under Axio.Scope A1 microscope (Carl Zeiss) equipped with AxioVs40 v4.8.2.0 software.

### Cellulose content

Cellulose amount was measured with a colorimetric method employing anthrone reagent [87]. The samples were incubated for 1 hour at 100°C in a mixture of nitric and acetic acid (1:8 v/v). After this samples were centrifuged, the supernatant discarded, the pellet washed twice with distilled water and then dissolved in 67% H_2_SO_4_ (v/v) and incubated for 1 h. To diluted samples cooled anthrone reagent was added. The cellulose content was measured spectrophotometrically at 620 nm.

### Total amount of pectin level

The measurement was conducted in three steps. The first one was to remove contamination form tissues by extracting the samples as follow, with 96% ethanol at 100°C, 80% ethanol at 80°C, chloroform:methanol solution (1:1 v/v) at 40°C and then acetone in the room temperature. After drying, the samples were hydrolyzed with concentrated H_2_SO_4_ in the ice bath. Diluted with water and centrifuged, the supernatant containing pectin was collected to the new tubes and further the amount of pectin was determined spectrophotometrically with the biphenyl method.

The hydrolyzate was supported in turn with 4 M sulfamic acid potassium sulfonate solution, pH = 1.6; Na_2_B_4_O_7_ in H_2_SO_4_, than incubated for 20 min at 100°C. Finally, m-hydroxybiphenyl was added to measure absorption at 525 nm. The results were given as an equivalent of glucuronic acid [68].

### The phenylpropanoid compounds analysis

To analyze metabolites from the phenylpropanoid pathway, the fibre samples were first subjected methanol extraction. Grounded to powder and weighed, 500 mg of fibre per sample was suspended in pure methanol, 5 ml, and extracted for 15 minutes in an ultrasonic bath.

•UPLC analysis of methanol extracts.

•After centrifugation, the supernatants were collected and the methanol extraction was repeated twice. Then the supernatants were collected, evaporated to dryness and the resultant pellets were resuspended in 2 ml of methanol for UPLC analysis.

•Alkali hydrolysis

•5 ml of 2 M NaOH was added to remaining pellets, which after vortexing were incubated for 48 hours at room temperature. After adjusting pH to 3, the collected hydrolyzates were extracted 3 times with ethyl acetate; the organic phases were collected, evaporated to dryness and then suspended in 2 ml of methanol for further analysis [68].

Quantification of the phenylpropanoides in both extracts was carried out on Waters Acquity UPLC system with a 2996 PDA detector, using an Acquity UPLC column BEH C18, 2.1 × 100 mm, 1.7 μm. The mobile phase was A = 0.1% formic acid and B = acetonitrile, in a gradient flow: 1 min at 95% A/5% B; 12 min gradient to 70% A/30% B; 15 min gradient to 0% A/100% B; and 17 min 95% A/5% B with a 0.1 ml/min flow rate. The detection of all phenolics was done at 320 nm.

### Mechanical properties

Tensile tests of ratted fibres were conducted by means of a computer driven Instron system (model 5542, High Wycombe, UK). The samples of the 10 mm gauge length were extended at a crosshead speed of 1 mm/min. The load displacement curve was recorded and used to evaluate fibre tensile parameters by means of the Bluehill 2 Software (Instron Co.).

Stiffness, measured as Young’ modulus, was calculated from the slope of a linear part of the load displacement curve (ΔF/Δx), using formulae E = (ΔF/Δx)/(L/A_cw_), where L was the gauge length and the A_cw_– the effective cell wall crossectional area. The maximum recorded load F_max_ per initial cross sectional area of the cell wall material, A_cw_, was used as a tensile strength measure, σ = F_max_/A_cw_. The cross section area of the cell wall material was evaluated using a gravimetric method [88] and formula A_cw_ = m/(L ∙d_cw_), where m is the weight of the fibre gauge length and d_cw_ -the cell wall material density assumed equal to 1540 kg m^-3^[82].

### FT-IR spectroscopic analysis

For infra-red spectroscopic analyze tissue samples were prepared as KBr pellet for mid infra-red spectrum or suspended in Njuol for far IR spectrum range. Measurements were done in a spectral range from 50 to 4000 cm^-1^ using a Biorad 575C FT–IR spectrometer.

### Scanning electron microscopy

For scanning electron microscopy 1 cm long pieces of flax mid-stalks were analyzed. Sample preparation was taken in two steps, coating specimens firstly with ultrathin layer of carbon, then secondly with pure silver using VEB Hochvakuum-Dresden B30.1 sputter. The specimens were probed using a scanning electron microscope Tesla BS 300 with copyright software at 15 kV.

### Statistical analysis

The data (±SD) was obtained from 3 to 5 samples per line and presented as the averages of independent replicates ± standard deviations. Statistical analysis was performed using Statistica 7 software (Statsoft, USA). The significance of the differences between the means was determined using Student’s t test, p value is given separately for each record, if not mentioned p value is above 0.05.

## Abbreviations

CAD: Cinnamyl alcohol dehydrogenase; SAD: Sinapyl alcohl dehydrogenase; HB(s): Hydrogen bond(s); 4CL: 4-coumarate-CoA ligase.

## Competing interests

The authors declare that they have no competing interests.

## Authors’ contributions

MP performed all the biochemical experiments, statistical analysis, lignin staining and SEM analysis, participated in the molecular genetic studies and phylogenetic analysis, phenotype analysis, field trial and flax processing execution, wrote the manuscript. AK carried out the UPLC analysis, participated in the design of the study and writing the manuscript. JZ performed mechanical analysis and participated in writing mechanical properties-related section of the manuscript. LD performed the infrared spectrophotometry analysis and participated in writing the IR-related section of the manuscript. JH designed FT-IR experiments and participated in the infra-red spectrophotometry analysis. MA participated in phenotype analysis, molecular genetic studies and field trial, flax processing execution. MS performed the pathogen resistance experiment. JS participated in study design and coordination. All authors read and approved the final manuscript.
